# Local allergic rhinitis: evolution of concepts

**DOI:** 10.1186/s13601-017-0174-7

**Published:** 2017-11-02

**Authors:** Cristoforo Incorvaia, Nicola Fuiano, Irene Martignago, Bruna L. Gritti, Erminia Ridolo

**Affiliations:** 1 Cardiac/Pulmonary Rehabilitation, ASST Pini/CTO, Milan, Italy; 2 Pediatric Immunology and Allergy Service, San Severo, Italy; 3 Medicine and Surgery Department, University of Parma, Via Gramsci 14, 43100 Parma, Italy

**Keywords:** Allergic rhinitis, Local allergic rhinitis, IgE production, Nasal mucosa, Nasal provocation test, Molecular allergy diagnostics

## Abstract

The discovery of an exclusive local production of IgE antibodies dates back to the 1970s, but only recently the pathophysiology of such phenomenon was deeply investigated, leading to the concept of local allergic rhinitis (LAR). Currently, LAR is defined by the occurrence, in patients with symptoms clearly suggesting allergic rhinitis but with negative results to common allergy testing, of allergen specific IgE in the nasal mucosa. Most studies investigating LAR were based on the development of rhinitis symptoms following nasal provocation test (NPT) with the suspected allergens, but such test may be performed by a number of options, none of them being as yet acknowledged and recommended in consensus document. On the other hand, also the mere detection of IgE in the nasal mucosa indicates, as for IgE measurement in blood or other tissues, allergic sensitization but cannot give the certainty of clinical allergy. Therefore, the combination of IgE detection in nasal mucosa and a positive result of NPT should be used to diagnose LAR. Recent data on the use for in vitro testing of molecular allergy diagnostics in place of whole allergen extracts suggest that this method could improve the sensitivity and specificity of laboratory tests, and an appraisal of the basophil activation test as a third level technique, to be implemented when the results of local IgE testing and NPT are uncertain, is currently ongoing.

## Background

The discovery of the exclusively local production of IgE antibodies in the nasal mucosa was made thanks to Huggings and Brostoff, who in 1975 reported that in patients with symptoms clearly suggesting allergic rhinitis (AR), but with negative results of allergy testing, IgE antibodies to *Dermatophagoides* could be found in nasal secretions [1]. Soon after, Johansson and Deuschl introduced a laboratory method to detect IgE in nasal secretions by which IgE were quantified in 52 of 60 analyzed secretions from patients with negative allergy tests [2] and in the late 1980s a method allowing to measure IgE directly in the nose was set up [3]. Indeed, in the following years the clinical significance of the local production of IgE was scarcely investigated. Only in 2010, Forester and Calabria reappraised the issue, reviewing the literature and proposing the concept of entopy (as opposed to atopy) to define the local production of IgE in the respiratory mucosa. However, they concluded that such concept was “both intriguing and controversial” due to the conflicting results of the studies then available [4]. In the latest years significant advances in the understanding of this allergic disorder were achieved, making hard to argue its existence as a clinical entity, but leaving room to define its epidemiological burden and the underlying pathophysiological mechanisms.

## The evidence supporting the actuality of local allergic rhinitis

The definition “local allergic rhinitis” (LAR) was first proposed by Rondón et al. [5] in a review analyzing the existing data. In particular, they had shown that nasal provocation test (NPT) with *Dermatophagoides pteronyssinus* was positive in 54% of patients with a diagnosis of nonallergic rhinitis (NAR) based on the negative results to common allergy testing [6]. The same authors recently reported in a 10-years follow-up study of a cohort of 176 patients with LAR of recent onset and 115 matched healthy controls that at the end of follow-up LAR patients experienced a significant and clinically relevant worsening of rhinitis with impairment of the quality of life. Importantly, conversion to AR (i.e. to systemic atopy) occurred in 9.7% of LAR patients compared to 7.8% of controls (*p* = 0.623). This defines LAR as a well-differentiated clinical entity with a low rate of progress to AR and a natural history towards worsening [7]. Indeed, these observations were based on the results of NPT with allergens, but such test is concerned by some limitations, that prevent to attribute to it a diagnostic role comparable, for example, to the placebo controlled food provocation test. NPT, though similarly based on the introduction in the nose of increasing amounts of allergens, may be interpreted through different methods, such as scoring the severity of nasal symptoms, by rhinomanometry, acoustic rhinometry and optical rhinometry. No consensus document gave recommendation on the best technique. For example, in recent studies a good specificity and accuracy of NPT, but a sensitivity lower than skin prick test (SPT), for house dust mites were reported using the symptoms scores [8]. While comparing different techniques measuring the nasal response to allergens, the most sensitive was the optical rhinometry [9]. Therefore, to obtain a firm diagnosis, the results of NPT need to be supported by other tools, particularly by detection of specific IgE in the nasal mucosa. Actually, in the study based on both methods, Rondón et al. [6] observed a positive response to NPT with *Dermatophagoides* in 54% of patients, but only 22% of patients had specific IgE in the nose. Fuiano et al. [10] reported that in a population of 192 patients with positive SPT results to aeroallergens, 111 with symptomatic AR and 81 asymptomatic, nasal IgE were detected in 77.5% of symptomatic patients, while only in 13.6% of asymptomatic patients. The same authors found that in children with rhinitis in the periods when *Alternaria* spores were present in the air, a positive result of nasal IgE and NPT was observed in 69.6% of patients, while positive SPT and NPT occurred in 26.8% of patients (*p* < 0.0001); this suggests that the association of NPT and nasal IgE has higher diagnostic capacity [11]. Such diagnostic value in *Alternaria* allergy was confirmed also in adults, in whom both positive allergen-specific NPT and specific IgE in nasal secretions were found [12].

## Issues in search of further definition

### Epidemiology of LAR

The actual prevalence of LAR is uncertain. LAR was long considered a rare disorder, until Rondón et al. [13] in 2012 reported in a group of 428 adult patients with rhinitis a LAR prevalence of 25.7%, compared with 63.1% of AR and 11.2% of NAR. The most frequently causative allergen in both forms was *D. pteronyssinus*. Similar ratios between LAR and AR were reported in a study on 219 elderly patients (mean age 65.8 years): 21% of patients had LAR and 40.2% had AR, *D. pteronyssinus* being the major culprit [14]. Such data were obtained on selected populations of patients with rhinitis, but the prevalence of LAR in the general population is still unexplored and warrants to be investigated in epidemiological surveys.

### Mechanisms underlying LAR

The pathomechanisms of AR are triggered by an inflammatory response in the nasal mucosa including an immediate IgE-mediated mast cell response and a late-phase response with recruitment of eosinophils, basophils and T cells expressing a Th2 cytokine profile, comprising interleukin (IL)-4 and IL-5. Recently, cytokines regulating the Th2 response, such as thymic stromal lymphopoietin, IL-25, and IL-33, were added as important factors [15]. The pathophysiology of LAR is scantly investigated, but a study on 40 patients with LAR from mites found that a NPT with the specific allergen elicited in 60% of patients immediate nasal symptoms and a significant increase of tryptase and eosinophil cationic protein (ECP), markers of mast cell and eosinophil activation respectively, in nasal lavage; 40% of patients had also a late-response, while no isolated late response was detected [16]. Campo et al. measured ECP in nasal lavages before and after NPT, with olive pollen and the major olive allergen Ole e 1, in three groups of patients with AR, LAR (both allergic to olive pollen) and healthy controls. Also, basophil activation test (BAT) with olive pollen and Ole e 1 was performed. ECP levels in nasal lavage were significantly higher after NPT in both AR and LAR compared with controls; all AR patients had a positive BAT to olive and 10/12 to Ole e 1, while 8/12 LAR patients had a positive BAT to olive and 4/12 to Ole e 1 [17]. These data show that the pathomechanisms of AR and LAR are similar but not identical. In particular, BAT is a promising diagnostic tool for LAR, but optimal allergen concentration to stimulate basophils remains to be defined for the single allergens associated to LAR. Another probe to explore the fine mechanisms working in AR and LAR is the response to allergen immunotherapy (AIT). The first placebo-controlled trial of subcutaneous AIT on LAR included 36 patients with mite allergy. Patients were randomized to receive active treatment or placebo for 24 months; symptoms, medication scores and medication-free days being the primary endpoints. AIT produced significant improvement versus placebo and after 12 months a significant increase in allergen tolerance was detected, with 50% of patients developing a negative NPT, along with significant increases of serum specific IgG4 antibodies [18]. This makes apparent that AIT also works on LAR, but this outcome must be confirmed by further trials with the most important allergens.

## Possible advances in diagnosing LAR

As discussed above, NPT alone cannot achieve a certain diagnosis of LAR. On the other hand, it was recently hypothesized that the nasal IgE production may represent a form of spontaneous immune response, based on a study on three small groups of subjects, one with AR, one with NAR, and healthy controls undergoing measurement of specific IgE to various allergens in nasal scrapings. Allergen-specific nasal IgE were detected in all groups (in 86.7, 33.3 and 50%, respectively), leading the authors to conclude that the presence of nasal IgE to allergens seems to be a non-specific phenomenon [19]. Indeed, this simply suggests that the detection of specific IgE in nasal mucosa does not escape the known limitation of IgE measurement in general, i.e. to indicate sensitization but not necessarily clinical allergy. Therefore, a double positive result of NPT and specific IgE in the nasal mucosa should be required to diagnose LAR. For IgE, the modern approach of component resolved diagnosis (CRD) has apparently a higher ability to identify the causative allergens. In fact, testing by immunoassay-biochip technology 112 different allergen components from 51 allergen sources in nasal secretions from patients with NAR no positive result was found [20]. This is divergent from the rate of positive results that is commonly reported when IgE to the whole allergen source are measured and suggests that CRD could be an in vitro test more sensitive and specific for diagnosis of LAR. Studies comparing the performance of CRD in respect to NPT are warranted, to establish whether molecular diagnostics could be used as preferred testing in patients with rhinitis and negative first level tests.

## Abbreviations

AIT: allergen immunotherapy
AR: allergic rhinitis
BAT: basophil activation test
CRD: component resolved diagnosis
ECP: eosinophil cationic protein
IL: interleukin
LAR: local allergic rhinitis
NPT: nasal provocation test
SPT: skin prick test
